# lncRNAs Are Involved in Sevoflurane Anesthesia-Related Brain Function Modulation through Affecting Mitochondrial Function and Aging Process

**DOI:** 10.1155/2020/8841511

**Published:** 2020-12-05

**Authors:** Yinyin Qu, Hongyi Li, Chengmei Shi, Min Qian, Ning Yang, Liwei Wang, Xingyu Gao, Cheng Ni

**Affiliations:** ^1^Department of Anesthesiology, Peking University Third Hospital, Beijing 100191, China; ^2^Department of Anesthesiology, National Cancer Center/National Clinical Research Center for Cancer/Cancer Hospital, Chinese Academy of Medical Sciences and Peking Union Medical College, Beijing 100021, China; ^3^Institute of Microelectronics, Chinese Academy of Sciences, Beijing 100029, China

## Abstract

Long noncoding RNAs (lncRNAs) play important roles in brain function modulation and neurodegenerative diseases. However, whether lncRNA regulations are involved in the mechanisms of perioperative neurocognitive disorders, especially in anesthesia-related brain dysfunction, remain unknown. Therefore, we explored the expression and regulation pattern profiles of lncRNAs in the hippocampus of aged rats after sevoflurane anesthesia. Three lncRNAs and 772 protein-coding genes were identified by microarray analysis and evidenced by in vitro and in vivo experiments as differentially expressed. Functional annotation and differentially expressed- (DE-) lncRNA-mRNA coexpression networks reveal that DE-lncRNAs are associated with mitochondrial dysfunction and oxidative stress, aging-related metabolism alterations, DNA damage, and apoptosis, as well as neurodegenerative features during sevoflurane anesthesia. These results suggest that lncRNAs play roles in general anesthesia-related brain function modulation during the perioperative context and provide insights into the lncRNA-related modulation mechanisms and targets.

## 1. Introduction

Every year, 66 million senior patients aged 65 and above undergo anesthesia and surgery all around the world, including 8.5 million patients with Alzheimer's disease (AD) [1]. Up to 40% of these patients may suffer from perioperative neurocognitive disorders (PND) [2], which include postoperative delirium (POD) and postoperative cognitive dysfunction (POCD) [3]. Increasing age, preoperative cognitive impairment, surgical trauma, and anesthesia may propose the onset of PND [2, 4–6]. Aging and related neurodegenerations have features of neuroinflammation and mitochondrial dysfunction, even amyloid plaques and neurofibrillary tangles, which damage the neural processes necessary for cognition [7, 8]. Surgery/anesthesia exposures contribute to the neuropathologic changes and cognitive decline in aged models [9]. Ours and related researches have indicated that inhaled general anesthesia is etiologically implicated in cognitive impairment in the aged brain [10, 11]. During the process, microglial inflammation [12], mitochondrial function deficits [13], and hippocampal synaptic plasticity modulation [14] could be the contributing pathological factors. However, the role of epigenetic modulation during general anesthesia-related brain function alterations remains unknown.

Long noncoding RNAs (lncRNAs) are noncoding RNAs with more than 200 nucleotides, which involve multiple epigenetic modulations. Increasing evidence suggests that lncRNAs play significant roles in the regulation of tissue homeostasis, oxidative stress, and metabolism [15, 16]. In the central nervous system (CNS), the fundamental roles of lncRNAs in various neurodegenerations are also becoming evident; representatives include *BACE1-AS*, *NEAT1_2*, and *Meg3*. These lncRNAs exert their regulation effect through various mechanisms such as interacting with mRNA (*BACE1-AS*) [17], binding to RNA-binding proteins (*NEAT1_2*) [18], or working as competing endogenous RNAs (*Meg3*) [19]. However, whether lncRNA regulations are involved in the mechanisms of PND, especially in general anesthesia-related brain function modulation, remain to be elucidated. Identification of candidate lncRNAs could provide insights into the mechanisms of the effects of anesthesia and efficient diagnostic strategies. Therefore, we focused on lncRNA and gene expression patterns in the hippocampus of aged rats after sevoflurane anesthesia. And we established functional annotation of differentially expressed- (DE-) lncRNAs and their potential target genes, as well as lncRNA-mRNA coexpression network to reveal the role of lncRNAs in sevoflurane anesthesia-related brain function modulation.

## 2. Materials and Methods

### 2.1. Animals and Anesthesia

The experiments were performed in accordance with the guide for the care and use of laboratory animals, and the protocol was approved by the Peking University Biomedical Ethics Committee Experimental Animal Ethics Branch (No. LA201412). As previously study described [20], adult male Sprague-Dawley rats, 18-month old, weighing between 550 and 600 g were used. The rats were maintained under standard housing conditions for 2 weeks before the experiment. Food and water were provided ad libitum. The rats were randomly assigned to the sevoflurane anesthesia or control group. For whole transcriptome microarray analysis, there were 3 rats in each group. For qPCR and other experiments, there were 6 rats or 6 cell samples in each group. Previous studies revealed that the minimum alveolar concentration (MAC) of sevoflurane for rats was between 2.4-2.7% [21]. In our study, rats in the sevoflurane anesthesia group received 2.5% sevoflurane in 100% oxygen for 4 hours in an anesthetizing chamber, and the control group received 100% oxygen at an identical flow rate for 4 h in an identical chamber. Sevoflurane and oxygen concentrations were monitored continuously (Datex, Tewksbury, MA, USA). The rats were breathing spontaneously at a stable ambient temperature. The rectal temperatures of the animals were maintained at 37 ± 0.5°C. This anesthesia protocol has been shown not to significantly alter the values of blood pressure and blood gas in the preliminary studies [20]. Anesthesia was terminated by discontinuing sevoflurane and placing animals in a chamber containing 100% oxygen until 20 minutes after the recovery of consciousness. The animals were then returned to individual home cages until sacrifice. Rats were sacrificed by decapitation. The brain tissues were removed rapidly, and the hippocampus was dissected out and frozen in liquid nitrogen.

### 2.2. Cell Culture and Treatments

C6 rat glioma cells (CLS Cat# 500142/p672_C6, RRID:CVCL_0194) were used in the studies. The cells were cultured in 6-well plates and grown at 37°C in an incubator with a humidified atmosphere with 5% CO_2_ in F-12K medium (Gibco™, Thermo Fisher Scientific, Waltham, MA, USA) as described in our previous study [22]. The medium was supplemented with 2.5% fetal bovine serum, 15% horse serum, 100 U/ml penicillin, and 100 *μ*g/ml streptomycin. S-100 production increases ten folds as cells grow from low density to confluency, which could induce abnormal gene expression regulation in the cells. As a result, about 18 h after seeding, we treated the cells when they reach approximately 70% confluency. The cells were randomly assigned to a treatment or control group. In the treatment group, the cells were treated in a sealed plastic box in a 37°C incubator, with 4.1% sevoflurane plus 21% O_2_ and 5% CO_2_, delivered from an anesthesia machine for 4 h as described by Dong et al. [23]. The cells in the control group received vehicle gas under the same condition in the absence of sevoflurane. A Datex-infrared gas analyzer (Puritan-Bennett, Tewksbury, MA) was used to continuously monitor the delivered concentrations of carbon dioxide, oxygen, and sevoflurane. Then, the cells were harvested and frozen in liquid nitrogen.

### 2.3. RNA Extraction and Quantification

Total RNAs were isolated from the hippocampi and cells using TRIzol reagent (Invitrogen, Carlsbad, CA), then digested with RNase-Free DNase to remove residual DNAs. The RNA concentrations were analyzed using the Nanodrop 2000 (Thermo Fisher Scientific), then total RNA (2 *μ*g) was reverse-transcribed using the GoScript™ Reverse Transcription System (Promega).

### 2.4. Quantitative Real-Time PCR (qRT-PCR)

We performed qRT-PCR in a total reaction volume of 10 *μ*l, including PowerUp SYBR® Green master mix (Thermo Fisher Scientific), 10 *μ*M PCR forward and reverse primers (Invitrogen, Carlsbad, CA, USA), and approximately 1.5 *μ*l of cDNA template on CFX96 Real-Time PCR Detection System (Bio-Rad, Hercules, CA, USA) according to the manufacturer's instructions. Primer sequences were obtained from the literature and checked for their specificity through in silico PCR. The forward and reverse primers of TC0900001760.rn.1 (NONCODE GENE ID: NONMMUG000308, Location Ch9: 36497845-9813) were 5′-AGCCCCAAAGTAAGACATTT-3′ and 5′-CCCCTTGAGATCACAATCAA-3′, of TC1300001223.rn.1 (NONCODE GENE ID: NONMMUG001518, Location Ch13: 28298352-300631) were 5′-TGGTAACCAACTACTTTCGG-3′ and 5′-AAACATGAGTGGAAGAGGTC-3′, of TC1800000859.rn.1 (NONCODE GENE ID: NONMMUG020388, Location Ch18: 73630496-1258) were 5′-CTCCATTCTCTTACTTGAC-3′ and 5′-CAGAGTGTACTAGGAAGCTC-3′, and of TC1400000903.rn.1 (NONCODE GENE ID: NONMMUG005150, Location Ch14: 84503818-11796) were 5′-GACTCATTCCAGCACAGTTA-3′ and 5′-CTTGAGGGAGAATAGCAGTC-3′ (These probe sets were designated according to mouse genes and located through sequence similarity alignment in rat genome). The forward and reverse primers of *Hif3a* were 5′-CACATGGACTGGGACCAAGACAGG-3′ and 5′-GTGTAGGCTGCTGGTGTGGAGTGT-3′, of *Prkcd* were 5′-CCATCTCATCTGTACCTTCC-3′ and 5′-CCATCCTTGTCCAGCATTA-3′, and of *Nfe2l2* were 5′-GCACATCCAGACAGACACCA-3′ and 5′-GGCTGGGAATATCCAGGGCA-3′. Amplification was carried out with an initial denaturation step at 95°C for 2 min, followed by 45 cycles of 95°C for 10 s, 55°C for 30 s, and 60°C for 30 s, then a final extension at 65°C for 2 min in 10 *μ*l reaction volume. After amplification, a melt curve was performed to make sure that none of the nonspecific products such as primer dimers were amplified. All reactions were run in duplicate, and the results were averaged from 6 independent studies. qPCR was quantified in two steps, firstly, *Actb* (*β*-actin) levels were used to normalize target gene levels (ΔCycle threshold (ΔCt) = Ct_target gene_–Ct_Actb_, target gene level = 2^−ΔCt^). Beta-actin was chosen as an internal control because previous studies confirmed that it was one of the most stable genes in the hippocampus of rats [24]. It also acts as a reliable endogenous control and has been widely used in the context of sevoflurane anesthesia in aged rats [25, 26]. Secondly, the target gene levels of the sevoflurane group were presented as the percentage of those of the control group, and 100% of the target gene levels referred to the control levels.

### 2.5. Affymetrix Whole Transcriptome Microarray Analysis and Functional Annotation

Whole transcriptome microarray analysis was performed using Clariom™ D Pico Assay, previously known as GeneChip™ Rat Transcriptome Array 1.0 (Affymetrix, Santa Clara, CA, https://www.thermofisher.com/order/catalog/product/902666). This array has a full coverage of the transcribed genome, including both coding and noncoding splicing variants. Briefly, isolated RNA (100 ng) was mixed with 1.5 *μ*l of Poly-A RNA control solution and subjected to reverse transcription. The obtained cDNA was used for in vitro transcription to prepare antisense RNA (aRNA) by incubation at 40°C for 16 h. Then, the aRNA was applied for the second round of sense cDNA synthesis using the WT Expression kit (Ambion, Austin, TX). The obtained cDNA was used for biotin labeling and fragmentation by Affymetrix GeneChip® WT Terminal Labeling and Hybridization. Biotin-labeled fragments of cDNA (5.5 *μ*g) were hybridized to the Affymetrix® Rat Transcriptome Array Strip (45°C/24 h), and up to 25 unique probes sequences were hybridized to a single transcript. Following hybridization, each array strip was washed and stained using the Fluidics Station of GeneChip® Scanner 3000 7G system (Affymetrix, Santa Clara, CA). The array strips were scanned using the Imaging Station of the GeneChip® Scanner 3000 7G system.

### 2.6. Mitochondrial Preparation

Tissue mitochondrial isolation kit (Beyotime Biotech, Shanghai, China) was used for mitochondrial preparation. The hippocampus was dissected out 3 h after anesthesia and placed in a freshly prepared cold mannitol solution encompassing 10 mM HEPES-potassium hydroxide, 70 mM sucrose, 0.1% (*w*/*v*) BSA, 200 mM d-mannitol, and 1 mM EDTA at pH of 7.4. Then, it was homogenized, the broken cell debris and nuclei were sedimentated by centrifugation at 600 × g and 4°C for 5 min, and the supernatant was carried out after centrifugation at 10,000 × g and 4°C for 10 min. The resulting mitochondrial pellets were suspended in the mannitol solution.

### 2.7. Reactive Oxygen Species (ROS) Assay

A ROS assay kit (Beyotime Biotech, Shanghai, China) with fluorescent probe DCFH-DA was used to measure the ROS level in the hippocampal mitochondria. Incubation of the hippocampal mitochondria suspensions (0.5 mg protein/ml) was carried out with 1.6 *μ*M 2′, 7′-dichlorofluorescin diacetate (DCFH-DA) at 37°C for 10 min. The fluorescence was measured with the Perkin Elmer LS-50B Luminescence fluorescence spectrophotometer (California, USA) at the excitation and emission wavelength of 488 and 525 mm, respectively. Then, the mean fluorescence intensities in excitation/emission were quantified and compared.

### 2.8. Mitochondrial Membrane Potential (MMP) Assay

An MMP assay kit (Beyotime Biotech, Shanghai, China) with fluorescent probe JC-1 was used to measure MMP of the hippocampal mitochondria. Incubation of the hippocampal mitochondria suspensions (0.5 mg protein/ml) was carried out with JC-1 reagent at 37°C for 15 min. The red fluorescence was measured at the excitation and emission wavelength of 525 and 590 mm, and green fluorescence was measured at the excitation and emission wavelength of 490 and 530 mm with the Perkin Elmer LS-50B Luminescence fluorescence spectrophotometer. The level of MMP was calculated by the ratio of red fluorescence to green fluorescence.

### 2.9. Statistical Analysis

Statistical analysis was performed with GraphPad Prism 5.0 software (http://graphpad.com, RRID: SCR_002798). The quantitative data are presented as the mean ± SD. In vivo and in vitro PCR validations were displayed with the violin plots, which depict the kernel probability density, and the width of the shaded area shows the proportion of the data located at that expression fold. Unpaired two-tailed Student's *t*-test was used to determine significant difference between the two groups. One-way ANOVA with Bonferroni's multiple comparison test was used to analyze significant differences between multiple groups. *p* < 0.05 was considered significant. The microarray analysis was performed by Expression Console Transcriptome Analysis Console Software. One-way ANOVA was applied. *p* value was adjusted with FDR method (Benjamini-Hochberg procedure). DE-lncRNAs were screened with *p* < 0.05 and ∣fold change | >1.5. Unsupervised two-way hierarchical clustering of DE-lncRNAs was illustrated in the heat map. The significance of GO and KEGG enrichment was calculated by the hypergeometric distribution and Fisher exact test, and a lower *p* value indicated that the specific term was more significantly enriched.

## 3. Results

**Figure 1:**
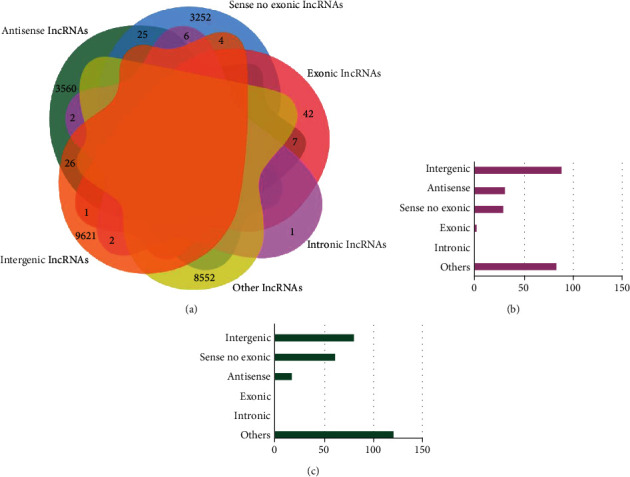
Distribution and expression of lncRNAs identified by whole transcriptome microarray analysis in the hippocampus of aged rats. (a) Venn distribution of lncRNAs. (b) Numbers of upregulated lncRNAs in different categories. (c) Numbers of downregulated lncRNAs in different categories.

The aged rats were randomly assigned to sevoflurane and control groups. The sevoflurane group received 4 h sevoflurane anesthesia, and the hippocampus was dissected out 3 h after anesthesia. In the hippocampus, 25204 lncRNAs were identified by whole transcriptome microarray analysis (Clariom™ D Pico Assay), which allows transcriptome profiling of both coding and noncoding genes using multiple databases. Since the functions and related mechanisms of lncRNAs could be speculated according to the classification based on the locations relative to protein-coding genes in the genome, the classification of these lncRNAs was analyzed. The results showed that 9654 lncRNAs (38.3%) were intergenic, 3620 lncRNAs (14.4%) were antisense, 3287 lncRNAs (13.0%) were sense no exonic, 50 lncRNAs (0.20%) were exonic, 11 lncRNAs (0.04%) were intronic, and 8582 lncRNAs (34.1%) were others (Figure 1(a)).

**Figure 2:**
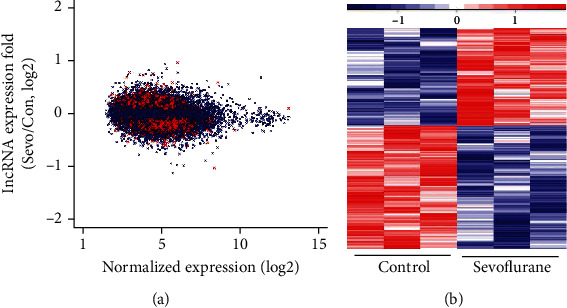
Differentially expressed lncRNAs after sevoflurane anesthesia in microarray analysis. (a) Ratio of gene expression in sevoflurane group to control condition (*y*-axis) and average expression of genes in sevoflurane group versus that in control condition (*x*-axis), presented as a Bland-Altman plot of our microarray analysis. Highlighted in red are lncRNAs with significant changes in expression (*p* < 0.05). (b) The heat map shows lncRNAs with significant changes from microarray analysis (*p* < 0.05).

The expression level of lncRNAs in the sevoflurane group versus that in the control group was presented as a Bland-Altman plot based on the microarray analysis, and 514 DE-lncRNAs were found and highlighted in red (*p* < 0.05, Figure 2(a)). Hierarchical cluster analysis showed a clear distinction in expression values of the DE-lncRNAs, in which 232 lncRNAs were found to be upregulated, and 282 lncRNAs were downregulated in the sevoflurane group (Figure 2(b)). Based on the abovementioned classification based on the genomic locations, the numbers of upregulated lncRNAs belonging to intergenic, antisense, sense no exonic, exonic, intronic, and others categories were 88, 31, 29, 1, 0, and 83, respectively (Figure 1(b)). Meanwhile, the numbers of downregulated lncRNAs that belong to the above six categories were 81, 18, 62, 0, 0, and 121, respectively (Figure 1(c)).

Taking ∣fold change | >1.5 as the cutoff, four lncRNAs were filtered out. Among them, NONMMUG000308, NONMMUG001518, and NONMMUG005150 were upregulated, and NONMMUG020388 was downregulated. These four lncRNAs all fell into the lincRNA category.

**Figure 3:**
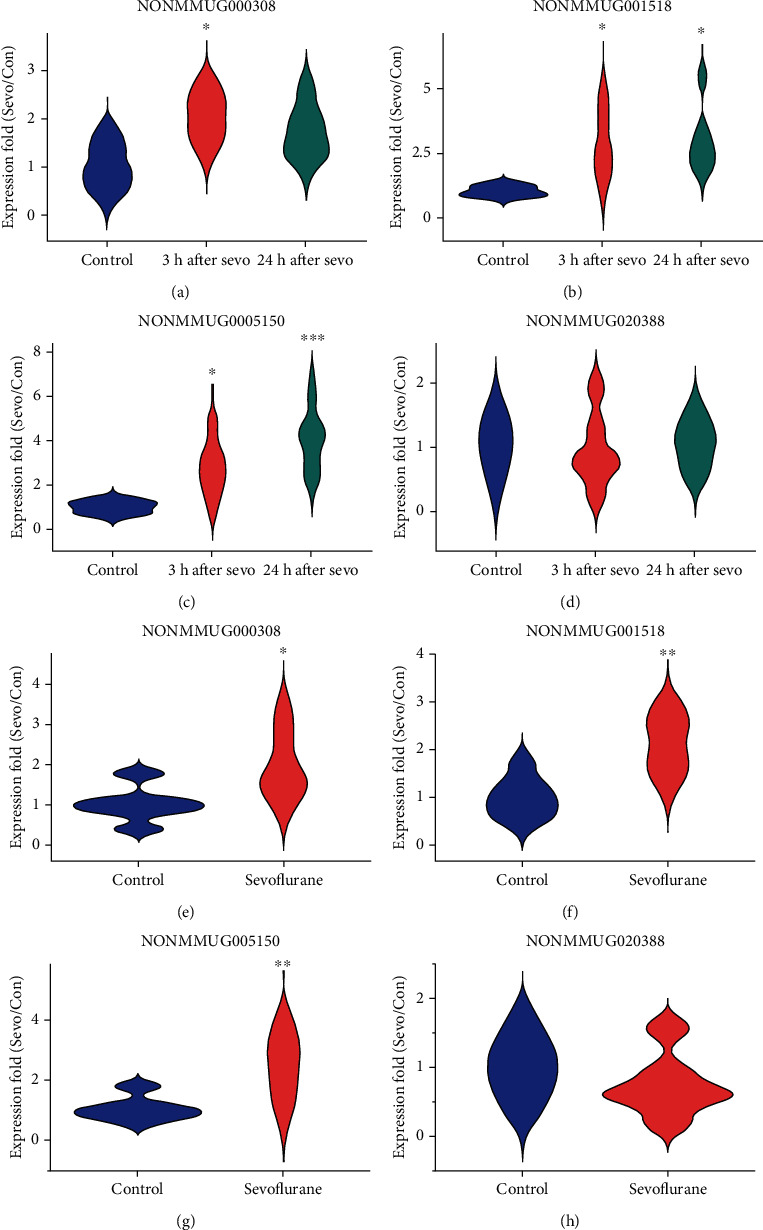
qPCR validation for differentially expressed lncRNA NONMMUG000308, NONMMUG001518, NONMMUG005150, and NONMMUG020388 in vivo (a–d) and in vitro (e–h). Gene expression was normalized and calculated using the 2^-*ΔΔ*Ct^ method. *n* = 6, ^∗^*p* < 0.05, ^∗∗^*p* < 0.01, and ^∗∗∗^*p* < 0.001 indicated differentiated samples compared with control condition.

To validate the microarray analysis results of four lncRNAs (NONMMUG000308, NONMMUG001518, NONMMUG005150, and NONMMUG020388), qPCR was employed. The aged rats were also assigned to sevoflurane and control groups. The sevoflurane group received 4 h sevoflurane anesthesia. Given that the expression of postoperative neurotoxicity and relative RNA transcription emerged at 3 h after surgery and could persist for 24 h [27, 28], the hippocampus was dissected out at 3 h and 24 h after anesthesia, respectively. Violin plots were used to illustrate the PCR results (*n* = 6, Figures 3(a)–3(h)). The violin plot diagrams depict the kernel probability density, and the width of the shaded area shows the proportion of the data located at that expression fold. The qPCR results were consistent with microarray results in the three candidate lncRNAs (NONMMUG000308, NONMMUG001518, and NONMMUG005150). For NONMMUG000308, the expression increased significantly at 3 h (2.04 ± 0.50 vs. 1.00 ± 0.44, *p* < 0.05), but not at 24 h (1.72 ± 0.54, *p* > 0.05) after anesthesia. For NONMMUG001518, the expression increased significantly at both 3 h and 24 h after anesthesia (2.87 ± 1.20 and 2.99 ± 1.32 vs. 1.00 ± 0.21, *p* < 0.05). For NONMMUG005150, the expression increased significantly at both 3 h (2.78 ± 1.25 vs. 1.00 ± 0.28, *p* < 0.05) and 24 h (4.03 ± 1.39, *p* < 0.001) after nesthesia. However, qPCR validation did not show a significant change for NONMMUG020388 (0.98 ± 0.29 and 1.03 ± 0.19 vs. 1.00 ± 0.23, *p* > 0.05). Furthermore, we performed an in vitro qPCR validation to confirm the uniformity of lncRNAs performance across a range of experimental conditions. The in vitro experiment was conducted in C6 rat glioma cells with control conditions and 4 h sevoflurane exposure. The results provided confirmatory evidence that NONMMUG000308 (1.98 ± 0.84 vs. 1.00 ± 0.45, *p* < 0.05), NONMMUG001518 (2.12 ± 0.58 vs. 1.00 ± 0.42, *p* < 0.01), and NONMMUG005150 (2.48 ± 1.05 vs. 1.00 ± 0.42, *p* < 0.01) in C6 cells are significantly upregulated after sevoflurane exposure, but no significant difference of NONMMUG020388 expression were found between two groups. Thus, the qPCR validation demonstrated good consistency between in vivo and in vitro models. As data quality parameters such as array *p* values and fold change may exert influence on the consistency of the two methods, we assume PCR validations across different experimental conditions are more reliable according to previous studies [29].

**Figure 4:**
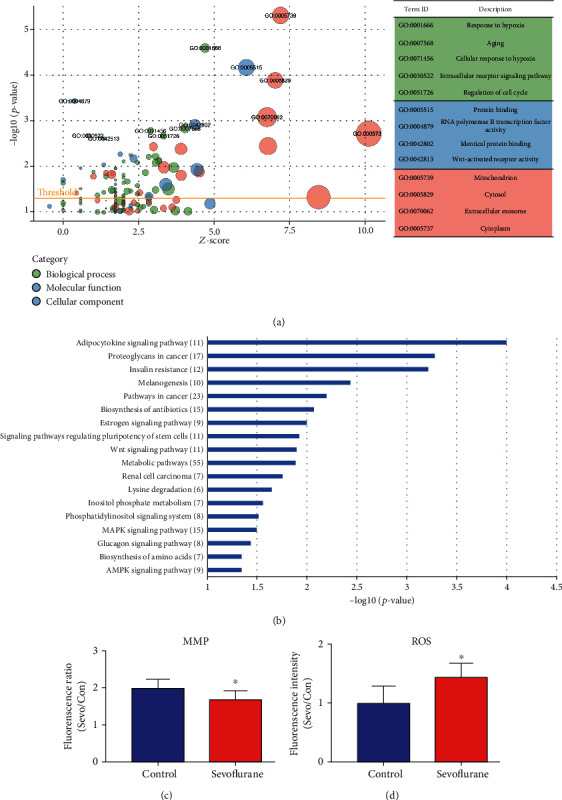
(a) Bubble plot of GO analysis of differentially expressed genes after sevoflurane anesthesia using the DAVID database. *y*-axis: the negative logarithm of *p* value. *x*-axis: *z* score of GO terms. The green, blue, and red bubbles indicate biological process, molecular function, and cellular component. *Z* score = (up − down)/√count. Up is the number of assigned genes upregulated (log2 (FC) > 0) in the data, and down is those downregulated (log2 (FC) < 0). (b) KEGG enrichment analysis of differentially expressed genes. *y*-axis: KEGG term descriptions. The numbers of differentiated genes enriched in each KEGG term were displayed in brackets. *x*-axis: the negative logarithm of *p* value of different KEGG terms. (c) MMP levels decreased after sevoflurane anesthesia, indicated by the ratio of red fluorescence to green fluorescence. (d) ROS levels increased after sevoflurane anesthesia, indicated by increased fluorescence intensity. ^∗^*p* < 0.05 indicated differentiated samples compared with the control condition.

Considering the three candidate DE-lncRNAs are all lincRNAs, which can positively or negatively adjust the expression of target genes, the expression of protein-coding genes in the hippocampus of aged rats was also detected by whole transcriptome microarray analysis. 772 DE-protein coding genes were identified after sevoflurane anesthesia (*p* < 0.05) with 608 genes upregulated and 164 downregulated. Then, Gene Ontology (GO) functional annotation and Kyoto Encyclopedia of Genes and Genomes (KEGG) pathway enrichment analysis were conducted for DE-protein coding genes using Database for Annotation, Visualization, and Integrated Discovery (DAVID, https://david.ncifcrf.gov). GO enrichment analysis contains three categories: biological process, molecular function, and cellular component. Hypergeometric distribution was used to determine whether a GO term is overrepresented after anesthesia. A lower *p* value indicated that the GO or KEGG term was more significantly enriched, and the results of DAVID GO analysis revealed that 44 GO terms of biological process, 16 terms of molecular function, and 18 terms of cellular component were significantly enriched after sevoflurane treatment (*p* < 0.05), respectively. A bubble plot to visualize enriched GO terms is shown in Figure 4(a). GO term circle in the upper part of the figure represents a relatively lower *p* value, while a circle with larger diameter indicates more genes were involved in a specific GO term. The top significantly overrepresented terms, sorted by *p* values in ascending order for biological process (green) were response to hypoxia, aging, cellular response to hypoxia, intracellular receptor signaling pathway, and regulation of cell cycle (*p* < 0.05); for molecular function (blue) were protein binding, RNA polymerase II transcription factor activity, identical protein binding, and Wnt-activated receptor activity (*p <0.05*); for cellular component (red) were mitochondrion, cytosol, extracellular exosome, and cytoplasm (*p* < 0.05).

Then, KEGG pathway analysis was employed to reveal involved molecular interaction, reaction, and relation networks after sevoflurane anesthesia.  Figure 4(b) highlighted 18 significantly enriched signaling pathways in our annotation using –log *p* > 1 as a threshold, including certain signaling pathways such as adipocytokine, estrogen, regulating pluripotency of stem cells, Wnt, MAPK, glucagon, and AMPK; as well as metabolic-related pathways such as metabolic pathways, lysine degradation, inositol phosphate metabolism, and biosynthesis of amino acids. Adipocytokine signaling pathway was the top significantly overrepresented pathway, and term metabolic pathways were associated with the most DE genes (55 DE-genes).

The mitochondrial membrane potential (MMP) is a factor determining the viability of mitochondria, thus plays a significant role in mitochondrial homeostasis [30]. It is also a driving force for ATP synthesis, and the loss of MMP could be the sign for apoptosis [31]. Thus, the MMP was assessed, and the results showed that compared with the control group, the level of MMP significantly decreased in the sevoflurane group (*p* < 0.05, Figure 4(c)). Mitochondrial dysfunction is the major cause of oxidative stress and reactive oxygen species (ROS) generation [32], so we measured the ROS level in the hippocampus of aged rats as well. The results showed that compared with the control group, the level of ROS increased significantly in the sevoflurane group (*p* < 0.05, Figure 4(d)). These results indicated that mitochondrial dysfunction, MMP decrease, and oxidative stress were involved in the pathophysiological processes after sevoflurane anesthesia, which are correlated with GO and KEGG analysis results.

**Figure 5:**
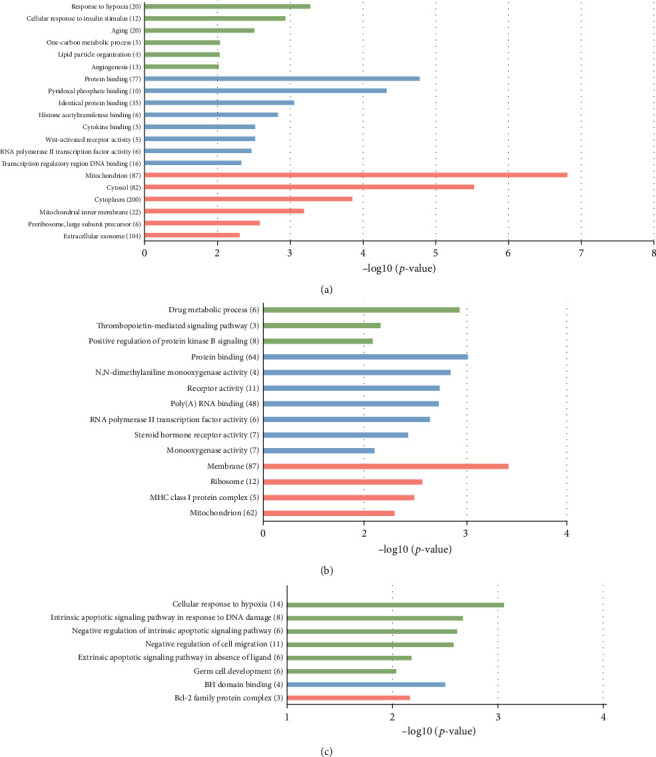
GO analysis of potential target genes of differentially expressed lncRNA NONMMUG000308 (a), NONMMUG001518 (b), and NONMMUG001518 (c). *y*-axis: GO terms of biological process, cellular component, and molecular function. The green columns indicate the biological process, the blue columns indicate molecular function, and the red columns indicate cellular component. The numbers of differentiated genes enriched in each term were displayed in brackets. *x*-axis: the negative logarithm of *p* value.

GO enrichment analysis revealed different patterns of potential target genes of the three upregulated lncRNAs after sevoflurane anesthesia. The results were ranked according to the negative logarithm *p* value, and the terms with -log *p* > 2 of each category were displayed. For the potential target genes of NONMMUG000308, response to hypoxia, protein binding, and mitochondrion were top significant enriched terms in biological process, molecular function, and cellular component, respectively (Figure 5(a)). Protein binding is an unspecific annotation which suggested target genes may interact with proteins like p53 or with one or more sites on receptor molecules like signaling receptors. For NONMMUG001518, drug metabolic process and protein binding were the top significant enriched terms in biological process and molecular function, and top cellular component term included membrane, ribosome, and mitochondria (Figure 5(b)). This indicated that products of the target genes may be found in cellular membrane, ribosome, or mitochondria. For NONMMUG005150, cellular response to hypoxia, BH domain binding, and Bcl-2 family complex were the top significant enriched terms in three categories (Figure 5(c)). Thus, potential target genes of three upregulated lncRNAs after sevoflurane anesthesia were enriched in oxidative stress, mitochondrion, metabolism, apoptosis, etc.

**Figure 6:**
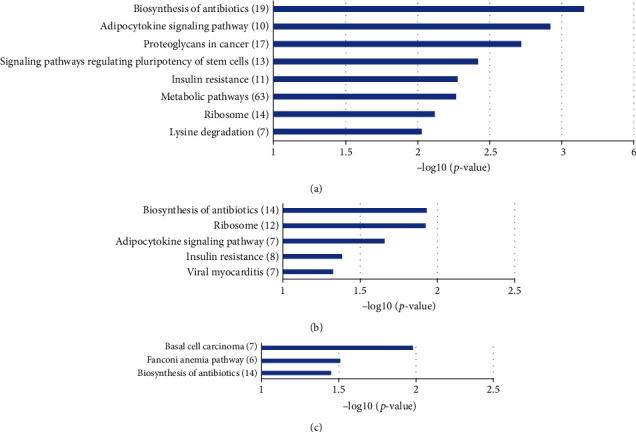
KEGG enrichment analysis of potential target genes of differentially expressed lncRNA NONMMUG000308 (a), NONMMUG001518 (b), and NONMMUG001518 (c). *y*-axis: KEGG terms descriptions. The numbers of differentiated genes enriched in each KEGG term were displayed in brackets. *x*-axis: the negative logarithm of *p* value of different KEGG terms.

KEGG pathway analysis revealed the signaling pathways of potential target genes of the three DE-lncRNAs after sevoflurane anesthesia. The results were ranked according to the negative logarithm of *p* value, and the terms with -log *p* > 1 were displayed. The signaling pathways of potential target genes of NONMMUG000308 and NONMMUG001518 are similar. Biosynthesis of antibiotics, signaling pathways of adipocytokine, and regulating pluripotency of stem cells, metabolic pathways were significantly enriched. Metabolic pathways including energy, carbohydrate, lipid, nucleotides, and amino acid metabolism were associated with the most potential target genes (63 DE-mRNAs, Figures 6(a) and 6(b)). For NONMMUG005150, the top enriched terms were basal cell carcinoma and Fanconi anemia pathway (Figure 6(c)). Pathways of carcinoma and anemia are characterized by abnormal proliferation and impaired response to DNA damage.

To explore the potential regulatory mechanism of the DE-lncRNAs, correlation analysis was carried out between the DE-lncRNAs and DE-protein coding genes. The DE protein-coding genes with Pearson correlation coefficient (PCC) > 0.85 and *p* < 0.05 were considered as the potential target genes of DE-lncRNAs. Pearson correlation is a traditional statistical metric widely used by previous studies to predict the function of lncRNAs by using information from coexpressed mRNAs [15, 33], and coexpressing genes are involved in related processes. Furthermore, the coexpression network of DE-lncRNAs and their potential target genes was established. Cytoscape 3.6.1 (http://www.cytoscape.org, RRID:SCR_003032) was used in network visualization, and the correlations with PCC > 0.85 for lncRNA-mRNA (protein-coding gene) pairs and PCC > 0.95 between mRNA-mRNA (protein-coding gene) pairs were selected.

**Figure 7:**
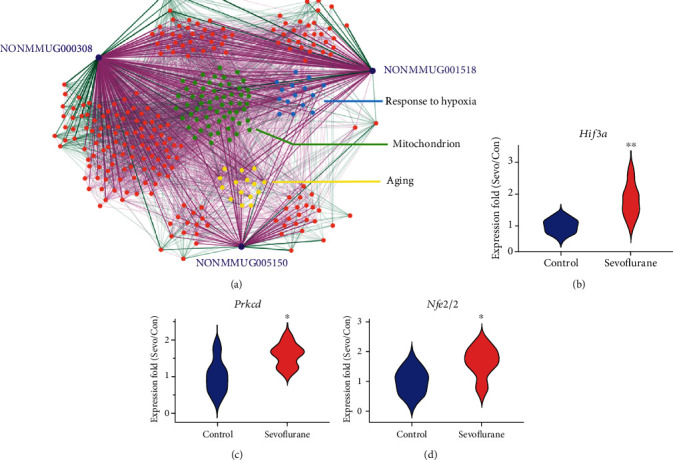
(a) LncRNA-mRNA coexpression network in the aged hippocampus after sevoflurane anesthesia. The purple nodes represent lncRNAs. The blue, green, yellow, and orange nodes represent gene-annotated GO terms response to hypoxia, mitochondrion, aging, and others. Coexpression pairs of lncRNA-mRNA whose Pearson correlation coefficient (PCC) > 0.85 and mRNA-mRNA pairs whose PCC > 0.95 were connected by straight lines. Positive correlations are shown in red lines, and negative correlations in green lines. Lower transparency of edges means higher PCC. qPCR validation for representative differentially expressed genes (b) *Hif3a* from term hypoxia, (c) *Prkcd* from term mitochondrion, and (d) *Nfe2l2* from term aging. *n* = 6, ^∗^*p* < 0.05, and ^∗∗^*p* < 0.01 indicated differentiated samples compared with the control condition.

The RNAs were displayed with nodes (three DE-lncRNAs and 296 mRNAs). Positive coexpression represented by red edges, negative correlation represented by green edges, and lower transparency of edges mean higher PCC. 342 positive and 53 negative lncRNA-mRNA pairs were displayed. NONMMUG000308 was coexpressed with 224 mRNA transcripts, 193 upregulated and 31 downregulated. NONMMUG001518 was coexpressed with 113 mRNA transcripts, 99 upregulated and 14 downregulated. And NONMMUG005150 was coexpressed with 58 mRNA transcripts, 50 upregulated and 8 downregulated. According to the above functional annotation, DE-lncRNAs were associated with oxidative stress and mitochondrial dysfunction, metabolic pathways, DNA damage, and apoptosis after sevoflurane anesthesia. Thus, the enriched term response to hypoxia, mitochondrion, and aging (with relatively lower *p* value) was selected as the representative terms. lncRNA coexpressed genes in these three terms were highlighted in blue, green, and yellow nodes, and other terms were highlighted in orange nodes. Genes that are highly correlated to each other are likely to be involved in the same pathway, and connections between protein-coding genes were also outlined to represent protein-protein interaction (Figure 7(a)).

Genes from term response to hypoxia, mitochondrion, and aging with relative high PCC with at least two lncRNAs were screened out. They included *Plat*, *Epas1*, *Prkcd*, *Agtrap*, *Tnfrsf1a*, *Hif3a*, and *Tgfbr3* in term response to hypoxia; *Gjb6*, *Prkcd*, *Nfel2l2*, and *Fgf2* in aging; as well as 16 genes (*Prkcd*, *Oxa1l*, etc) in term mitochondrion. Then, three representative DE-protein coding genes were selected and validated by qPCR (*Hif3a* from term hypoxia, *Prkcd* from term mitochondria, and *Nfe2l2* from term aging). Compared with control conditions, the expression of *Hif3a* (1.82 ± 0.52 vs. 1.00 ± 0.21, *p* < 0.01), *Prkcd* (1.58 ± 0.31 vs. 1.00 ± 0.44, *p* < 0.05), and *Nfe2l2* (1.66 ± 0.52 vs. 1.00 ± 0.41, *p* < 0.05) in the hippocampus increased significantly at 3 h after anesthesia (*n* = 6, Figures 7(b)–7(d)). These results also indicated a good consistency between qPCR and microarray analysis results.

As lncRNA NONMMUG000308, NONMMUG001518, and NONMMUG005150 were differentially expressed after sevoflurane anesthesia and might play regulatory roles through oxidative stress and mitochondrial dysregulation, aging-related metabolic alterations, DNA damage, apoptosis, and neurodegenerative features according to the present results, we named NONMMUG000308, NONMMUG001518, and NONMMUG005150 as *sevoflurane associated noncoding RNA* (*Sancr*) *1*, *2*, and *3*, respectively.

## 4. Discussion

The present study screened three significantly DE-lncRNAs and multiple DE-protein coding genes in the hippocampus of aged rats after sevoflurane anesthesia by microarray analysis. In vitro and in vivo experiments verified the analysis results. Functional annotation with GO and KEGG databases showed that the top target overrepresented terms of lncRNAs included response to hypoxia, aging, and mitochondrion. A network of DE-lncRNAs and their potential target genes were established, and these DE-lncRNAs are named as *Sancr 1*, *2*, and *3*, respectively.

As previous studies indicated that the hippocampal pathophysiological changes play important roles in the processes of POCD [13, 34], the hippocampus was selected for the present study. Cell culture [35], animal [36], and human [37] data indicate that inhaled anesthetics could accelerate the development of aging and related neurodegenerations including apoptosis, A*β* generation, and tau phosphorylation. Combined with the present results, we infer that anesthesia also plays a role in the development of aging and related pathophysiological processes.

Accumulating evidences indicate the role of lncRNAs in the setting of oxidative stress, altered metabolism, and aging process, and they act as transcriptional factors or bind with target genes to exert functions. Several lincRNAs were differentially expressed in the hippocampus of both transgenic models and patients of AD [38, 39], and the dysregulation of lincRNAs played key roles in the intricate regulation of CNS development and disorders [40, 41]. For example, lncRNA *BC200* is a transcript found in the brain with a function of inhibiting translation initiation. *BC200* levels in cortical areas are reduced by above 60% in normal aging, in contrast, they are significantly upregulated in AD and paralleled with the deterioration of the disease [42]. lncRNA *BACE1-AS* concentrations were increased in AD subjects. It could change the secondary or tertiary structure of BACE1 through a mechanism affecting RNA duplex formation and so that increases its stability [17], and BACE1 is a crucial enzyme responsible for oligomer production in AD. Thus, we assumed that NONMMUG000308, NONMMUG001518, NONMMUG005150, and NONMMUG020388 regulate the expression level of target genes, which participates in the mechanism of sevoflurane-associated brain function modulation.

Since monitoring during anesthesia indicated that the rats did not suffer from hypoxia, and hippocampal MMP loss and ROS generation were observed during sevoflurane anesthesia, and emerging evidence revealed that HIFs expression can be induced by aberrant factors independent of oxygen levels [43, 44]. Hif3a and Epas1 (also called Hif2a) coding proteins are members of hypoxia-inducible factor (HIF) family, which are crucial modulators of transcriptional response to hypoxic stress [45, 46]. We assumed that *Sancrs* regulated perioperative oxidative stress and related genes, such as *Epas1* and *Hif3a*, transcriptional level, like lncRNA *Neat2* [47]. Previous studies showed *Hif3a* mRNA expression can be regulated at the transcriptional level via hypoxia response elements [48] or posttranscriptional level via miRNA [49]. Gene Ontology annotations related to *Hif3a* include DNA-binding transcription factor activity and transcription coactivator activity, which are included in protein binding and consistent with the enriched terms in molecular function annotation of *Sancr* target genes.

The results indicated that *Nfe2l2*, *Mthfd1l*, *Akt1*, and *Atg5* were possible targets of three DE-lncRNAs in metabolic pathways. In GO term aging, Nfe2l2 was identified as a regulator of autophagy in degrading intraneuronal aggregates [50]. Nfe2l2 encoded transcription factor NRF2, which is the chief regulator to defense against stress in mammalian cells. NRF2 activation alleviates cognitive deficits of AD models through modulation of oxidative stress [51]. MTHFD1L, an enzyme in the folate cycle that is transcriptionally activated by NRF2, plays an essential role in maintaining proper mitochondria function [52]. AKT1 could induce mitophagy due to reactive oxygen species [53]. Inhibition of mitochondrial permeability transition pore opening decreases ROS and increases MMP [54], and superoxide dismutase, glutathione peroxidase, and catalase levels could be affected during the process. The results also indicate that metabolism-related gene terms are affected by anesthesia. Combined with the present results, we propose that *Sancrs* interact with target genes and affect the metabolic process in the brain.

*Sancrs* were correlated to *Prkcd* expression, which was associated with the transcriptional regulation of p53 in response to DNA damage [55]. Dysregulation of DNA damage response is associated with neurodegenerative disorders [56], and the linkage between lncRNAs and neuron apoptosis has also been investigated in neurodegenerative diseases [57]. Both PKC*δ* and p53 are associated with the apoptotic mechanisms in the mitochondria through Bcl-2 family proteins modulation and to provide mitochondrial outer membrane permeabilization [58]. Damage-induced lncRNA can create a feedback loop to amplify DNA damage signaling or interact with DNA damage RNAs through RNA-RNA pairs [59]. Thus, it is possible that *Sancrs* regulate genes like *Prkcd* or interact with other RNAs in this process and induced cell death. Our functional annotation results show that BH domain binding and the intrinsic mitochondrial apoptotic pathway Bcl-2 family complex are top significant enriched terms, which indicates *Sancrs* could be involved in intrinsic apoptosis in the hippocampus after anesthesia [60].

Our results show that *Sancrs* were highly coexpressed with *Plat*, and they could play a crucial role in perioperative ischemia-related diseases. Tissue plasminogen activator (encoded by *Plat*) is involved in the breakdown of blood clots. Genetic variants of *Plat* and plasminogen activator inhibitor type-1 combinations have been suggested to be the risk factors for stroke [61], and gene interactions could change the susceptibility of the disease. Disorders like amyotrophic lateral sclerosis, AD, and Parkinson's disease have also been linked to reduced NRF2 levels [62, 63]. Combined with the results, we speculate that *Sancrs* bind with *Nfe2l2*, then accelerate stroke and neurodegenerative processes [64].

## 5. Conclusion

In the present investigation, we identified three DE-lncRNAs, *Sancr 1*, *2*, and *3* in the hippocampus of aged rats after sevoflurane anesthesia. *Sancrs* regulate mitochondrial dysfunction and oxidative stress, aging-related metabolism alterations, DNA damage, and apoptosis, as well as stroke and neurodegenerative features in the hippocampus, which play roles in anesthesia-related cognitive function modulation during perioperative context. And similar to the mechanism of AD, a complex network of deregulated and multitasking lncRNAs together interacts with these pathophysiological mechanisms. These results provide evidence for the lncRNA regulation network in anesthesia-related brain function modulation, which could be the understanding from an epigenetic perspective. However, detailed regulation pathways and correlated factors are unclarified. Further work should therefore investigate the accurate mechanisms and provide brain protection strategies through epigenetic perspective during the process.

## Data Availability

The data of this study are openly available in Gene Expression Omnibus at https://www.ncbi.nlm.nih.gov/geo, with reference number GSE139220.
